# Prevalence and trends of polypharmacy among HIV-positive and -negative men in the Multicenter AIDS Cohort Study from 2004 to 2016

**DOI:** 10.1371/journal.pone.0203890

**Published:** 2018-09-11

**Authors:** Deanna Ware, Frank J. Palella, Kara W. Chew, M. Reuel Friedman, Gypsyamber D’Souza, Ken Ho, Michael Plankey

**Affiliations:** 1 Department of Infectious Diseases, Georgetown University Medical Center, Washington, District of Columbia, United States of America; 2 Feinberg School of Medicine, Northwestern University, Chicago, Illinois, United States of America; 3 David Geffen School of Medicine, University of California Los Angeles, Los Angeles, California, United States of America; 4 Infectious Diseases and Microbiology, Graduate School of Public Health, University of Pittsburgh, Pittsburgh, Pennsylvania, United States of America; 5 Bloomberg School of Public Health, Johns Hopkins University, Baltimore, Maryland, United States of America; 6 Department of Medicine, University of Pittsburgh, Pittsburgh, Pennsylvania, United States of America; San Antonio Military Medical Center, UNITED STATES

## Abstract

Rates of aging-related comorbidities, which require targeted medications to treat, have been shown to be increased among persons living with HIV compared with uninfected counterparts. Polypharmacy is generally defined as the concurrent use of 5 or more medications. We investigated polypharmacy prevalence for non-HIV medications over a 12-year period among HIV-positive and -negative participants in the Multicenter AIDS Cohort Study. Information regarding non-HIV medication use, HIV status, age, race/ethnicity, enrollment period, and medication insurance was obtained on 3,160 participants from semiannual visits between 2004 and 2016. Polypharmacy was defined as taking 5 or more non-HIV medications since the last health care visit. Generalized estimating equation models with repeated measures were produced overall and by HIV status to examine polypharmacy. The unadjusted prevalence of polypharmacy across all study visits was 18.6% and was higher among HIV-positive participants (24.4%) compared with HIV-negative participants (11.6%) (*P* < .0001). Among the 50 years and older age group, HIV-positive and HIV-negative participants had increases in polypharmacy over the observation period, from 38.4% to 46.8% (*P* = .0081) and from 16.7% to 46.0% (*P* < .0001), respectively. Among participants younger than 50, polypharmacy among HIV-positive participants remained stable (18.9% in 2004 to 17.3% in 2016; *P* = .5374) but increased among HIV-negative men (5.6% to 20.4%; *P* < .0001). After adjusting for age, race/ethnicity, and medication insurance, HIV-positive participants had a higher prevalence of polypharmacy than HIV-negative participants (25.3% vs 18.7%; *P* < .0001). Older age, white race, and having medication insurance coverage were also associated with greater polypharmacy. A convergence of polypharmacy prevalence was observed between HIV-positive and -negative participants at the end of observation. HIV-positive status was associated with an increased likelihood of polypharmacy, after adjusting for age, race/ethnicity, enrollment period, medication insurance, and study visit. Over time, polypharmacy prevalence increased among all participants, with converging rates between HIV-positive and -negative participants by the end of the observation period.

## Introduction

Nearly half of persons living with HIV (PLWH) in the United States are older than 50 years of age [1]. In the general US population, adults experience increasing rates of comorbidities as they age [2–4]. PLWH develop these comorbidities at a higher rate than those without HIV [5–7]. While the number of drugs that comprise a modern antiretroviral therapy regimen has diminished over time, aging PLWH increasingly require more non-HIV medications to treat chronic age-related comorbidities [8]. Polypharmacy is generally defined as the concurrent use of 5 or more non-HIV medications [9]. One recent study found that men living with HIV take a median of 13 medications, including medications for HIV infection [10], underscoring the prevalence of polypharmacy among PLWH. Established risks of polypharmacy include increased serious adverse drug events, organ system injury, hospitalization, decreased medication adherence, and mortality [11].

Several factors contribute to polypharmacy among PLWH. Lower-income individuals have a greater risk of polypharmacy due to higher rates of non-HIV comorbidities and yet, having medication insurance coverage has been shown to correlate with excessive polypharmacy [10]. With the number of non–AIDS-related deaths currently outnumbering AIDS-related deaths among PLWH in the United States, PLWH older than 50 may have an enhanced susceptibility to polypharmacy due to comorbidities that result, at least in part, from ongoing immune dysfunction, decreased organ system reserve, and chronic inflammation [1, 11, 12]. In a Swiss HIV cohort study, multivariable hazard ratios for myocardial infarction, stroke, bone fractures (not due to trauma), diabetes, osteoporosis, and non–AIDS-defining malignancies were greater for PLWH older than 65 years vs younger PLWH [13].

To our knowledge, there is limited evidence comparing polypharmacy longitudinally between PLWH and HIV-negative individuals. In the general US population, polypharmacy has almost doubled, from 8.2% in 1999–2000 to 15% in 2011–2012 [14]. Among HIV-specific cohorts, polypharmacy prevalence rates ranged from 23% to 39%, with studies noting higher polypharmacy among PLWH compared with those who were HIV negative [15–18]. While these studies provide insight into the trends of polypharmacy in HIV-infected and uninfected populations, these studies were cross-sectional and limited to mostly older populations. To this end, we sought to examine the prevalence of polypharmacy in a population of HIV-positive and -negative men who have sex with men (MSM) in the Multicenter AIDS Cohort Study (MACS) over a 12-year period. We aimed to (1) determine the prevalence of polypharmacy and use of select non-HIV medications and (2) investigate risk factors associated with polypharmacy. We hypothesized that polypharmacy would be associated with HIV-positive status, after adjusting for age, enrollment period, medication insurance coverage, race/ethnicity, and study visit. Among HIV-positive participants, we hypothesized that polypharmacy would be associated with older age, having detectable plasma HIV RNA (viral load), and lower CD4+ T-lymphocyte cell count.

## Materials and methods

### Study population

The MACS is a prospective cohort study of the natural and treated history of HIV among MSM in 4 US regions: Baltimore, Maryland/Washington, DC; Chicago, Illinois; Los Angeles, California; and Pittsburgh, Pennsylvania/Columbus, Ohio. Since its inception in 1984, a total of 6,972 HIV-positive and HIV-negative MSM have been enrolled in the study over 3 periods: 4,954 in 1984–1985; 668 in 1987–1991; and 1,350 in 2001–2003. MACS participants attend semiannual clinic visits that involve an Audio Computer-Assisted Self-Interview and a standardized clinical examination, in which medical history data and biological specimens are collected and stored. The MACS study design has been described elsewhere [19–21]. Detailed information regarding HIV and non-HIV medication use has been collected at every visit since the beginning of the study. Questionnaires are available at www.aidscohortstudy.org. Institutional review boards at Johns Hopkins University, Northwestern University, University of California Los Angeles, and University of Pittsburgh approved the protocol, and written informed consent was obtained from all study participants. This analysis included 3,160 (1,715 HIV-positive/1,445 HIV-negative) men who participated in the MACS at any visit between October 2004 and April 2016.

### Outcome measures

#### Non-HIV medications

As part of routine data collection and processing during each semiannual visit, information regarding self-reported non-HIV medications was collected and given drug codes that were created for use within the MACS. Drug codes were generated using guidelines from the World Health Organization Anatomical Therapeutic Chemical index. Non-HIV medications were grouped into general drug classifications: cholesterol-lowering, antihypertensive, diabetes, hepatitis C virus, hepatitis B virus (including antiretroviral treatments), steroid, hormone, anticancer, antidepressant, tranquilizer, aspirin, antibiotic, and unclassified drugs. For this analysis, additional drug classifications were created for drug codes that were originally “unclassified.” These new classes were antihistamine, appetite suppressant, anti-anginal, anticoagulant, antidiarrheal, antifungal, antiulcer, central nervous system stimulant, digestive/biliary, dopamine, herbal supplement, muscle relaxant, nonsteroidal anti-inflammatory, opioid, substance abuse treatment, antituberculosis, and vitamins. Medications with a prevalence of use less than 5% were combined into an “other” medication category. There were a total of 30 medication classifications. Non-HIV medications included prescription and nonprescription drugs and excluded recreational drugs.

#### Polypharmacy

The total number of non-HIV medications taken at each visit was calculated. Polypharmacy was defined as a dichotomous indicator of 5 or more non-HIV medications taken since participants’ last visit. Routinely used and as-needed medications were treated similarly. All HIV antiretroviral medication was excluded from the polypharmacy count.

### Covariates

#### Sociodemographic characteristics

Race/ethnicity at baseline was categorized as non-Hispanic white, non-Hispanic black, and other. Enrollment was classified into early recruitment (1987–1991) and later recruitment (2001-current). Age at each visit was calculated from the self-reported date of birth and date at visit and was categorized as younger than 50 and older than or equal to 50 years. Race/ethnicity and enrollment were collected on entry into the MACS. Age was allowed to vary with time. As a result, when a participant aged from younger than 50 years to 50 years or older, they were recategorized.

#### Comorbidities

Selected comorbidities were examined and included high blood pressure (systolic blood pressure ≥ 140 mm Hg or diastolic blood pressure ≥ 90 mm Hg or the use of medications to treat high blood pressure), diabetes (fasting glucose ≥ 126 mg/dL or the use of medications to treat diabetes), liver disease (aspartate aminotransferase or aminotransferase > 150 UL), kidney disease (estimated glomerular filtration rate using Chronic Kidney Disease Epidemiology Collaboration equation < 60 mL/min/1.73 m^2^ or urine protein-to-creatinine ratio ≥ 200), and dyslipidemia (total cholesterol ≥ 200 mg/dL, low-density lipid ≥ 130 mg/dL, high-density lipid < 40 mg/dL, triglyceride ≥ 150 mg/dL, or the use of medications to treat dyslipidemia) [22].

#### Medication insurance coverage and health care visits

Current medication insurance coverage was defined using self-reported responses to the question, “*Do you have health insurance that covers the cost of medications*?*”* For each participant, the number of visits to a physician’s office, emergency department, or other health care clinic was calculated at each visit.

#### HIV-related risk factors

HIV status (HIV positive/HIV negative) was assessed using enzyme-linked immunosorbent assay with confirmatory Western blot on all MACS participants at their initial visit and at every visit for those who were HIV negative. HIV-positive participants included all men who were identified as such at baseline and those who seroconverted during study observation. CD4-positive T-lymphocyte cell counts/mm^3^ (CD4) and plasma HIV RNA levels (viral load) were collected among participants with HIV. CD4 and HIV RNA viral load were dichotomized into less than 500 cells/mm^3^ and greater than or equal to 500 cells/mm^3^ and detectable/undetectable (based on the lower detection level of the assay used at the visit), respectively.

#### Antiretroviral therapy

Antiretroviral therapy (ART) use was dichotomized into “yes” and “no” at each visit at which a participant reported use. Therapy type was categorized into (1) none, (2) monotherapy, (3) combination therapy, or (4) HAART. ART adherence was assessed using the question, “*On average*, *how often did you take your medication as prescribed*?*”* Response choices included: (1) 100% of the time, (2) 95% to 99% of the time, (3) 75% to 94% of the time, and (4) less than 75% of the time [23].

### Statistical analysis

Descriptive statistics were generated for the outcome measures and for each of the covariates at the index visit using frequencies/percentages and medians/interquartile ranges (IQRs) where appropriate. The index visit was defined as a participant’s first visit during the observation period between 2004 and 2016. Adjusted prevalence rates of polypharmacy and select medication classifications (antidepressants, antihypertensive, cholesterol lowering, and steroids) were calculated, stratified by age (<50/≥50 years) and HIV status (HIV positive/HIV negative), and graphically plotted. To longitudinally examine polypharmacy, generalized estimating equation models with repeated measures were produced overall and reproduced within each HIV status group. Covariates were considered for inclusion in the model based on a priori knowledge of their association with polypharmacy and included sociodemographic characteristics, medication insurance, and enrollment period. Race/ethnicity and enrollment period were time stable variables, while age, medication insurance, HIV-related risk factors, and ART use were longitudinally analyzed. Each covariate was independently tested for its association with polypharmacy and included in the model if the *P* value was less than .10. The final overall and HIV status–stratified models were adjusted for age, race/ethnicity, medication insurance coverage, enrollment period, and HIV status (in overall model only). In the HIV-positive–only model, there was additional adjustment for viral load, CD4, ART use, and ART adherence. Adjusted prevalence ratios (aPRs) along with their corresponding 95% CIs were generated. Missing values were considered to be random and were dropped from the analysis. Statistical significance was set at the *P* < .05 level. Statistical analyses were performed using SAS version 9.4 (SAS Institute Inc., Cary, North Carolina, USA).

## Results

### Descriptive statistics

Most participants identified as white non-Hispanic (60.0%) and 54.3% were recruited during the first enrollment period. The median follow-up time was 12.0 years (IQR, 5.0 to 12.5). Demographic characteristics stratified by HIV status are reported in Table 1. Evaluation of selected aging-related comorbidities at the participants’ index visit revealed prevalence rates of high blood pressure, diabetes, dyslipidemia, liver disease, and kidney disease of 17.8%, 4.9%, 30.0%, 0.1%, and 2.3%, respectively. At the index visit, HIV-negative participants had higher prevalence rates of high blood pressure (20.3% vs 15.7%) and dyslipidemia (32.8% vs 27.6%) than HIV-positive participants, while the latter had greater prevalence of diabetes (5.2% vs 4.5%), liver disease (1.0% vs 0.1%), and kidney disease (2.9% vs 1.6%). HIV-positive participants reported more medication insurance coverage at their index visit than HIV-negative participants at 86.0% and 77.0%, respectively. The overall median number of health care visits (physician’s office, emergency department, or health clinic) in the 6 months preceding the index visit was 2 (IQR, 1 to 5), with HIV-positive participants having more visits than their HIV-negative counterparts (3 vs 1; *P*>.0001). There was no statistically significant difference in health care visits by HIV status at end of the observation period. Among HIV-positive participants at their index visit, 48.2% had an undetectable viral load, 62.3% were at least 95% adherent to their ART medications, 47.9% had a CD4 count of greater than or equal to 500 cells/mm^3^, and were prescribed a median of 3 (IQR, 0 to 3) ART medications. Nearly one-third of HIV-positive participants (28.8%) reported no ART use (Table 1). The median number of non-HIV medications used by participants at the index visit was 2 (IQR, 1 to 4), with a higher number among HIV-positive participants compared with those who were HIV negative (3 vs 1; *P* < .0001). The prevalence of polypharmacy at the index visit was 18.6% and was higher in HIV-positive participants (24.4%) than HIV-negative participants (11.6%) (*P* < .0001). Demographic characteristics stratified by enrollment period are shown in Table 2. Men recruited earlier in the study were older (median age, 51 vs 39 years), mostly non-Hispanic white (85.2% vs 31.9%), and reported more medication insurance coverage (85.0% vs 78.3%) compared with later-enrollment participants.

**Table 1:** Characteristics of MACS participants by HIV status at index visit.

	HIV Negative	HIV Positive	All Participants
	(n = 1445)	(n = 1715)	(n = 3160)
**Follow-up, median (IQR), y**	11.5 (3.0–12.5)	12.5 (8.5–12.5)	12.0 (5.0–12.5)
**Age, median (IQR), y**	49 (41–56)	45 (38–51)	46 (39–53)
**Race/ethnicity, n (%)**			
Non-Hispanic white	991 (68.6%)	904 (52.7%)	1895 (60.0%)
Non-Hispanic black	303 (21.0%)	511 (29.8%)	814 (25.8%)
Other	151 (10.5%)	300 (17.5%)	451 (14.3%)
**Enrollment period, n (%)**			
Early recruitment (1987–1991)	921 (63.7%)	743 (43.3%)	1715 (54.3%)
Late recruitment (2001–2003)	524 (36.3%)	972 (56.7%)	1445 (45.7%)
**Prevalence of comorbidities, n (%)**			
Hypertension	293 (20.3%)	269 (15.7%)	562 (17.8%)
Diabetes	65 (4.5%)	89 (5.2%)	154 (4.9%)
Dyslipidemia	474 (32.8%)	473 (27.6%)	947 (30.0%)
Liver disease	2 (0.1%)	18 (1.0%)	20 (0.1%)
Kidney disease	23 (1.6%)	49 (2.9%)	72 (2.3%)
**Medication insurance coverage, n (%)**			
Yes	1112 (77.0%)	1475 (86.0%)	2587 (81.7%)
No	288 (19.9%)	193 (11.3%)	481 (15.2%)
Missing	45 (3.1%)	47 (2.7%)	92 (2.9%)
**No. of health Care visits, median (IQR)**	1 (0–3)	3 (1–6)	2 (1–5)
**HIV therapy type, n (%)**			
None	-	493 (28.8%)	493 (28.8%)
Monotherapy	-	7 (0.4%)	7 (0.4%)
Combination therapy	-	110 (6.4%)	110 (6.4%)
HAART	-	1073 (62.6%)	1073 (62.6%)
Missing	-	32 (1.9%)	32 (1.9%)
**HIV viral load, n (%)**			
Detectable	-	709 (41.3%)	709 (41.3%)
Undetectable	-	734 (42.8%)	734 (42.8%)
Missing	-	272 (18.9%)	272 (18.9%)
**ART adherence, n (%)**			
100%	-	436 (25.4%)	436 (25.4%)
95%-99%	-	633 (36.9%)	633 (36.9%)
75%-94%	-	112 (6.5%)	112 (6.5%)
<75%	-	31 (1.8%)	31 (1.8%)
Missing	-	503 (29.3%)	503 (29.3%)
**CD4**^**+**^ **count (cells/mm**^**3**^**)**^,^ **n (%)**			
< 500	-	678 (39.5%)	678 (39.5%)
≥ 500	-	821 (47.9%)	821 (47.9%)
Missing	-	216 (12.6%)	216 (12.6%)
**No. of ART medications, median (IQR)**		3 (0–3)	3 (0–3)
**No. of non-HIV medications, median (IQR)**	1 (1–3)	3 (2–4)	2 (1–4)
**Prevalence of selected medication types, n (%)**			
Antidepressants	277 (19.2%)	451 (26.3%)	728 (23.0%)
Cholesterol-lowering	198 (13.7%)	318 (18.5%)	516 (16.3%)
Steroids	57 (3.9%)	241 (14.1%)	298 (17.4%)
Antihypertensive	249 (17.2%)	276 (16.1%)	525 (16.6%)
**Polypharmacy, n (%)**			
≥5 Non-HIV medications	168 (11.6%)	419 (24.4%)	587 (18.6%)
<5 Non-HIV medications	1277 (88.4%)	1296 (75.6%)	2573 (81.4%)

Abbreviations: ART, antiretroviral therapy; IQR, interquartile range.

**Table 2:** Characteristics of MACS participants by enrollment at index visit.

	Early Enrollment	Late Enrollment	All Participants
	(n = 1664)	(n = 1496)	(N = 3160)
**Follow-up, median (IQR), y**	12.5 (9.5–12.5)	10.5 (2.0–12.5)	12.0 (5.0–12.5)
**Age, median (IQR), y**	51 (47–57)	39 (32–45)	46 (39–53)
**Race/ethnicity, n (%)**			
Non-Hispanic white	1418 (85.2%)	477 (31.9%)	1895 (60.0%)
Non-Hispanic black	148 (8.9%)	666 (44.5%)	814 (25.8%)
Other	98 (5.9%)	353 (23.6%)	451 (14.3%)
**HIV status, n (%)**			
Positive	743 (44.7%)	972 (65.0%)	1715 (54.3%)
Negative	921 (55.4%)	524 (35.0%)	1445 (45.7%)
**Prevalence of comorbidities, n (%)**			
Hypertension	401 (24.1%)	161 (10.8%)	562 (17.8%)
Diabetes	103 (6.2%)	51 (3.4%)	154 (4.9%)
Dyslipidemia	616 (37.0%)	331 (22.1%)	947 (30.0%)
Liver disease	9 (0.5%)	11 (0.7%)	20 (0.1%)
Kidney disease	44 (2.6%)	28 (1.9%)	72 (2.3%)
**Medication insurance coverage, n (%)**			
Yes	1415 (85.0%)	1172 (78.3%)	2587 (81.7%)
No	176 (10.6%)	305 (20.4%)	481 (15.2%)
Missing	73 (4.4%)	19 (1.3%)	92 (2.9%)
**No. of health care visits, median (IQR)**	2 (1–5)	2 (1–4)	2 (1–5)
**HIV therapy type, HIV only (n = 1715)**			
None	164 (22.1%)	329 (33.9%)	493 (28.8%)
Monotherapy	3 (0.4%)	4 (0.4%)	7 (0.4%)
Combination therapy	83 (11.2%)	27 (2.8%)	110 (6.4%)
HAART	465 (62.6%)	608 (62.6%)	1073 (62.6%)
Missing	28 (3.8%)	4 (0.4%)	32 (1.9%)
**HIV viral load, n (%), HIV only (n = 1715)**			
Detectable	263 (35.4%)	446 (45.9%)	709 (41.3%)
Undetectable	308 (41.5%)	426 (43.8%)	734 (42.8%)
Missing	172 (23.2%)	100 (10.3%)	272 (18.9%)
**ART adherence, n (%), HIV only (n = 1715)**			
100%	203 (27.3%)	233 (24.0%)	436 (25.4%)
95%-99%	307 (41.3%)	326 (33.5%)	633 (36.9%)
75%-94%	47 (6.3%)	65 (6.7%)	112 (6.5%)
<75%	14 (1.9%)	17 (1.8%)	31 (1.8%)
Missing	172 (23.2%)	331 (34.1%)	503 (29.3%)
**CD4**^**+**^ **count (cells/mm**^**3**^**)**^,^ **n (%), HIV only (n = 1715)**			
< 500	294 (39.6%)	384 (39.5%)	678 (39.5%)
≥ 500	295 (39.7%)	526 (54.1%)	821 (47.9%)
Missing	154 (20.7%)	62 (6.5%)	216 (12.6%)
**No. of ART medications, median (IQR), HIV only**	3 (2–4)	3 (0–3)	3 (0–3)
**No. of non-HIV medications, median (IQR)**	3 (1–4)	2 (1–3)	2 (1–4)
**Prevalence of selected medication types (unadjusted), n (%)**			
Antidepressants	427 (25.7%)	301 (20.1%)	728 (23.0%)
Cholesterol-lowering	400 (24.0%)	116 (7.8%)	516 (16.3%)
Steroids	217 (13.0%)	81 (5.4%)	298 (17.4%)
Antihypertensive	366 (22.0%)	159 (10.6%)	525 (16.6%)
**Polypharmacy, n (%)**			
≥5 Non-HIV medications	382 (23.0%)	205 (13.7%)	587 (18.6%)
<5 Non-HIV medications	1282 (77.0%)	1291 (86.3%)	2573 (81.4%)

Abbreviations: ART, antiretroviral therapy; IQR, interquartile range.

#### Adjusted prevalence of Non-HIV medications

**Fig 1:**
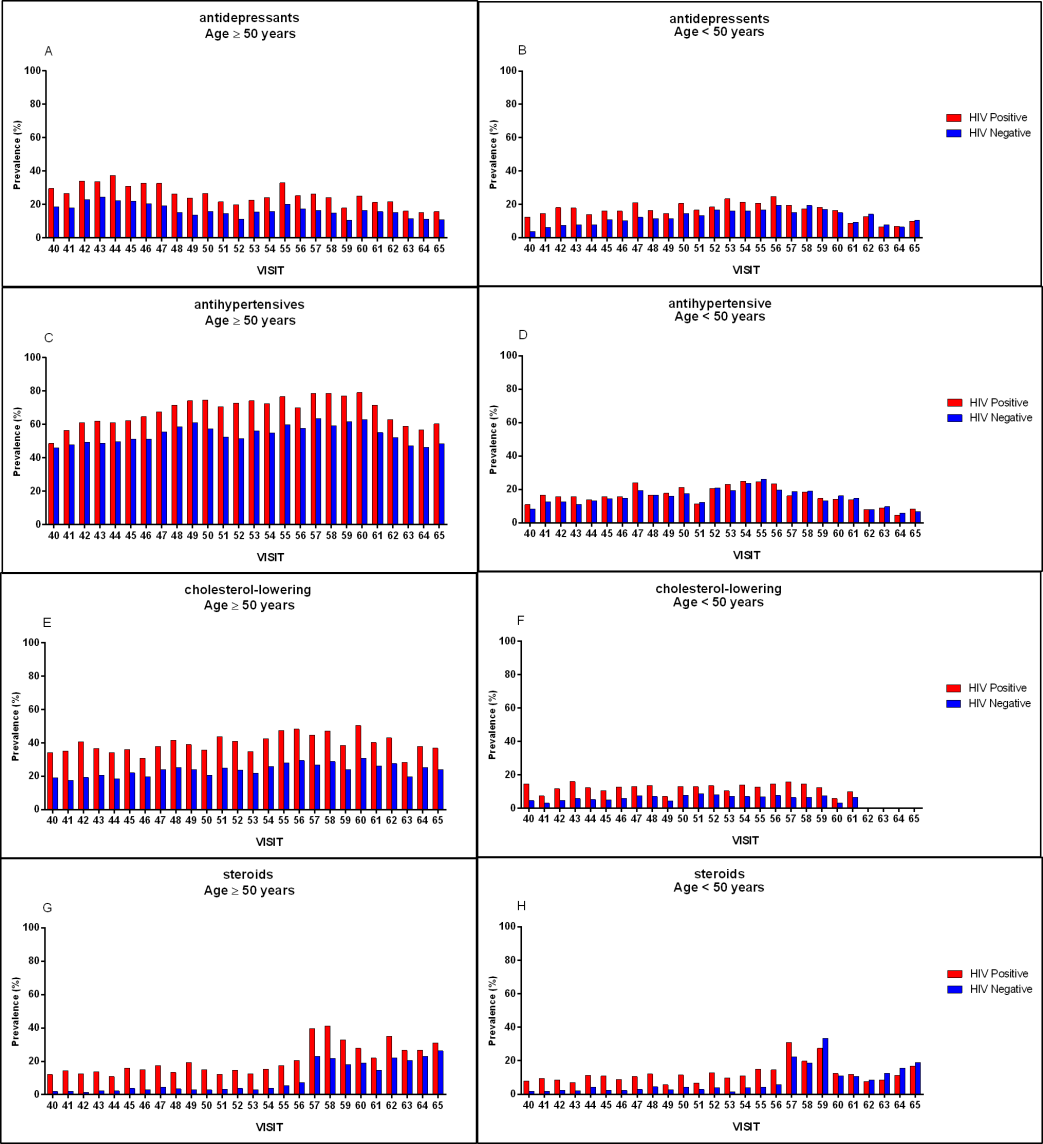
Prevalence of select medications by age, HIV status, and visit. Figure shows adjusted prevalence of antidepressant, antihypertensive, cholesterol-lowering, and steroid medication use among those 50 years and older (A, C, E, and G, respectively), and among those younger than 50 years old (B, D, F, and H, respectively).

Overall, the adjusted prevalence rates of antidepressant, antihypertensive, cholesterol-lowering, and steroid medication use were 22.1%, 30.6%, 29.2%, and 14.5%, respectively. HIV-positive participants reported more antidepressant (25.5% vs 18.6%; *P* < .0001), cholesterol-lowering (31.0% vs 27.4%; *P* < .0001), and steroid (19.1% vs 9.7%; *P* < .0001) use than HIV-negative persons. There were similar prevalence rates of antihypertensive medication use between HIV-positive and -negative participants (30.6% vs 30.6%; *P* = .8760). Other differences were observed by age and by HIV status (Fig 1). Participants older than 50 and HIV-positive participants had higher prevalence of use of the aforementioned medications than persons younger than 50 and HIV negative. Overall, the use of antihypertensive, cholesterol-lowering, and steroid medications increased over time (*P* < .0001), while antidepressant use remained relatively stable. The changes in prevalence of use of these medications from 2004 to 2016 are graphically represented in Fig 1.

#### Risks of polypharmacy

**Fig 2:**
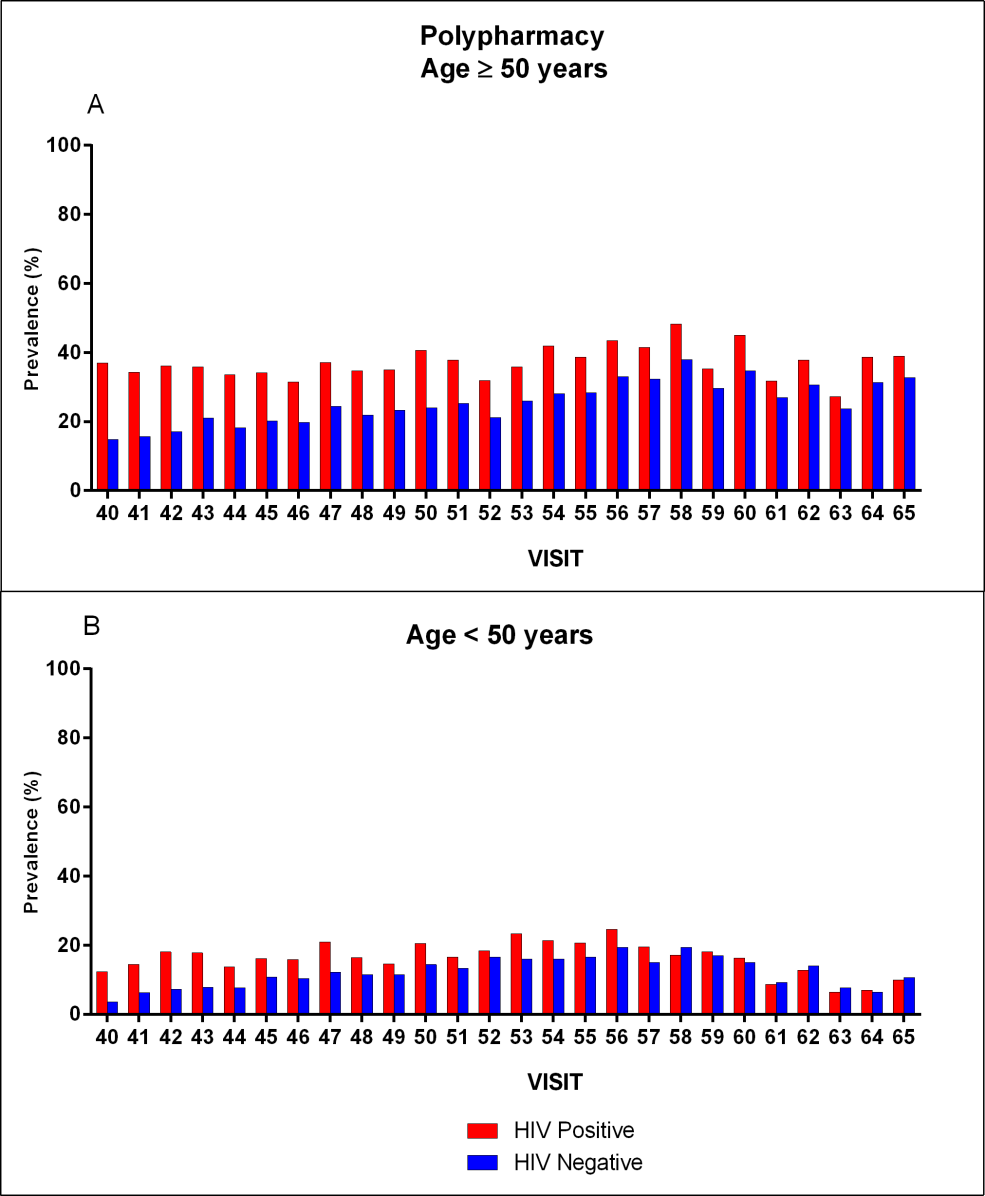
Prevalence of polypharmacy by age, HIV status, and visit. (A) Polypharmacy prevalence among HIV-positive and -negative participants 50 years and older. (B) Polypharmacy prevalence among HIV-positive and -negative participants younger than 50 years old.

We observed an increase in polypharmacy prevalence regardless of age and independent of HIV status from 2004 to 2016. In the 50 and older age group, HIV-positive and HIV-negative participants demonstrated increases in polypharmacy over time, from 38.4% to 46.8% (*P* = .0081) and from 16.7% to 46.0% (*P* < .0001), respectively. Among participants younger than 50 years, polypharmacy in HIV-positive participants remained stable, from 18.9% in 2004 to 17.3% in 2016 (*P* = 0.5374) but increased among HIV-negative men, from 5.6% to 20.4% (*P* < .0001) during the same period (Fig 2). By 2016, no significant differences in polypharmacy were apparent by HIV status regardless of age group. After adjusting for age, race/ethnicity, medication insurance coverage, and enrollment period, polypharmacy prevalence across all visits was 25.3% and 18.7% among HIV-positive and HIV-negative participants, respectively (*P* < .0001). Several risk factors for polypharmacy were identified. HIV-positive status (aPR, 1.36; 95% CI, 1.26 to 1.46), age 50 or older (aPR, 1.61; 95% CI, 1.47 to 1.76), insured (aPR, 1.32; 95% CI, 1.16 to 1.49), or enrolled earlier (aPR, 1.26; 95% CI, 1.14 to 1.40) were positively associated with polypharmacy, while other race (aPR, 0.86; 95% CI, 0.75 to 0.99) and non-Hispanic black (aPR, 0.73; 95% CI, 0.65 to 0.82) were associated with decreased odds of polypharmacy (Table 3). Similar risk factors were identified when considering HIV-positive and -negative participants separately. Additionally among HIV-positive participants, ART medication use was associated with increased risk of polypharmacy (aPR, 1.13; 95% CI, 1.12 to 1.18; Table 3).

**Table 3:** Adjusted risk factors for polypharmacy in participants.

	Adjusted Prevalence Ratios (95% CI)		
	Overall	HIV Positive	HIV Negative
**HIV status**			
Positive	1.36 (1.26 to 1.46) ^a^	-	-
Negative	Referent	-	-
**Age**			
≥ 50 y	1.61 (1.47 to 1.76) ^a^	1.22 (1.13 to 1.33) ^a^	1.56 (1.32 to 1.84) ^a^
< 50 y	Referent	Referent	Referent
**Race/ethnicity**			
Non-Hispanic black	0.73 (0.65 to 0.82) ^a^	0.83 (0.74 to 0.92) ^a^	0.94 (0.77 to 1.16)
Other	0.86 (0.75 to 0.99) ^a^	1.04 (0.93 to 1.16)	0.82 (0.63 to 1.08)
Non-Hispanic white	Referent	Referent	Referent
**Medication insurance coverage**			
Yes	1.32 (1.16 to 1.49) ^a^	1.14 (1.01 to 1.27) ^a^	1.90 (1.59 to 2.28) ^a^
No	Referent	Referent	Referent
**Enrollment period**			
Early recruitment (1987–1991)	1.26 (1.14 to 1.40) ^a^	1.08 (0.99 to 1.18) ^a^	1.42 (1.18 to 1.72) ^a^
Late recruitment (2001–2003)	Referent	Referent	Referent
**Viral Load**	-		-
Detectable	-	0.97 (0.92 to 1.02)	-
Undetectable	-	Referent	-
**CD4**^**+**^ **count (cells/mm**^**3**^**)**			
< 500	-	0.97 (0.91 to 1.02)	-
≥ 500	-	Referent	-
**ART adherence**			
95%-99%	-	1.04 (0.99 to 1.10)	-
75%-94%	-	0.98 (0.89 to 1.09)	-
<75%	-	0.95 (0.80 to 1.14)	-
100%	-	Referent	-
**ART medication use**	-	1.15 (1.12 to 1.18) ^a^	-

Abbreviation: ART, antiretroviral therapy; ^a^ Statistically significant; *p* values were calculated using generalized estimating equation models.

## Discussion

In this study, the overall prevalence of polypharmacy increased among MACS participants over a 12-year period and was driven by increases in the use of medications to treat aging-related chronic comorbidities, prominently including antihypertensive and cholesterol-lowering drugs. As expected, HIV-positive and older participants (≥50 years) carried the greatest burden of polypharmacy, with HIV-positive participants 50 years and older having the highest rates. Having medication insurance coverage was associated with increased polypharmacy. Participants who were non-Hispanic black and other race were less likely to experience polypharmacy than non-Hispanic white participants. Earlier MACS enrollment was also positively associated with polypharmacy. Participants enrolled during the earlier recruitment period were on average older and reported higher rates of medication insurance, therefore greater rates of polypharmacy were expected, regardless of HIV status. HIV-related clinical factors (viral load and CD4 cell count) and self-reported ART adherence among HIV-positive participants did not demonstrate a statistically significant association with polypharmacy. However, ART medication use itself was positively associated with increased polypharmacy. Unexpectedly, polypharmacy prevalence by HIV status was similar in both age groups toward the end of the observation period.

Our findings corroborate the trend of increased polypharmacy among HIV-positive and -negative populations [14–18]. Recently, Justice et al reported that polypharmacy was common in both HIV-positive and -negative individuals, and they found similar factors associated with polypharmacy, such as older age, white race, and HIV-positive status [17]. Since the introduction of effective ART, HIV-positive individuals experience markedly extended survival [24], and hence experience non-HIV chronic comorbidities. These comorbidities often occur at higher rates and earlier ages than among HIV-negative persons [8]. Our data revealed conflicting results regarding selected aging-related comorbidity rates by HIV status. HIV-negative participants more often reported diabetes, dyslipidemia, and high blood pressure than HIV-positive persons; however, this could have been in part due to HIV-negative participants having been older. Nevertheless, we found that use of medications used to treat these diseases (diabetes, dyslipidemia, and hypertension) was slightly greater among HIV-positive participants. This could be due, in part, to HIV-negative participants reporting lower rates of medication insurance coverage and health care visits overall. However, polypharmacy rates converged between HIV-positive and -negative participants at the end of the observation period. HIV-negative participants had a lower number of health care visits in 2004 compared with HIV-positive participants. By 2016, the numbers of health care visits by HIV status were similar. In analyses restricted to HIV-positive participants, we found that having a detectable viral load, CD4 less than 500, and ART adherence were not associated with a greater likelihood of polypharmacy, while ART use was associated with polypharmacy; this finding could be related to increased dyslipidemia and cardiovascular disease reported in association with ART use among HIV-positive persons [25–26].

Our study was subject to several limitations. We relied on self-reported medication use, which presented the possibility of recall bias. Short-term medication use, such as antibiotics, may have been underreported, especially if taken for short periods of time. Finally, this study used a nonrandom convenience sample and was restricted to HIV-positive and -negative MSM in 4 major metropolitan regions. Therefore, these results may not be generalizable to other MSM in the general population. Despite these limitations, there were some important strengths in this analysis including the ability to evaluate the long-term characterization of polypharmacy in a cohort for which standardized collection of medication use was semiannually recorded, in contrast to previously published polypharmacy studies, which were cross-sectional.

### Conclusion

In the MACS, polypharmacy was more prevalent among participants who were HIV positive, 50 years and older, enrolled during the earlier recruitment period, and had medication insurance coverage. Increased rates of comorbidities among the participants drove the main finding of increasing rates of polypharmacy over time. We also observed a convergence of polypharmacy prevalence between HIV-positive and -negative participants by the end of the observation period, which could be explained by increasing health care visits by HIV-negative participants. Further research will be needed to elucidate determinants of increased polypharmacy and to explore the appropriateness of prescribing practices that may contribute to polypharmacy among PLWH.
